# Dostoevsky, The Brothers Karamazov, and Epilepsy

**DOI:** 10.7759/cureus.38602

**Published:** 2023-05-05

**Authors:** James G Gamble

**Affiliations:** 1 Orthopedic Surgery, Stanford University School of Medicine, Lucile Packard Children's Hospital, Stanford, USA

**Keywords:** epilepsy, brothers karamazov, illness and creativity, psychogenic non-epileptic seizures, dostoevsky

## Abstract

Fyodor Mikhailovich Dostoevsky was a brilliant nineteenth-century Russian novelist who had a seizure disorder that influenced his life and his creativity. His novels explore issues of love, faith, doubt, morality and reflect his personal experience with epilepsy. He was a keen observer of familial psychodynamics. *The Brothers Karamazov *(1880)was Dostoyevsky’s longest and last novel, completed just a few months before his death from a pulmonary hemorrhage, most likely related to his life-long habit of cigarette smoking. In this novel, he explores the subtility of interpersonal relationships and the psychopathology within the Karamazov family and how one of the three brothers, Smerdyakov, uses psychogenic non-epileptic seizures as an alibi to get away with the perfect crime of patricide.

## Introduction and background

Fyodor Mikhailovich Dostoevsky (1821 - 1881) was a brilliant, nineteenth-century Russian novelist who suffered from epilepsy. His letters and diaries poignantly describe the anxiety he experienced because of the seizures. Dostoevsky’s personal experiences with epilepsy coupled with his broad understanding of human psychology has contributed to some of the most engaging literature of the nineteenth century: Notes from Underground (1864), Crime and Punishment (1866), The Idiot (1868-69), The Possessed (1872), The Brothers Karamazov (1879-80). He wrote thirteen novels, and his characters are so richly developed that over a century later we can feel a personal acquaintance and easily become engrossed in their social and psychological struggles. Dostoevsky explores issues of faith, doubt, morality, and love through the literary thoughts and actions of his characters.

The Brothers Karamazov was Dostoevsky’s last and longest novel, completed just a few months before his death from a pulmonary hemorrhage, most likely as a complication of his life-long habit of cigarette smoking. In this novel, Dostoevsky used his characters to delve into two important philosophical questions. First, what is the human predicament? Second, how should we live our lives? [1]. The purpose of this review is to discuss the influence of epilepsy on Dostoevsky’s life and writing, and to focus on his use of epilepsy in one of his characters, Smerdyakov from The Brothers Karamazov, as a demonstration of his deep and personal understanding of the physical and psychological dimensions associated with a seizure disorder.

## Review

Summary of the plot and the characters

The plot of The Brothers Karamazov centers on the events leading up to, and the aftermath of, the murder of Fyodor Pavlovich Karamazov, the 55-year-old father of the brothers. Fyodor Pavlovich is a miserable alcoholic, an ill-natured man, and a terrible father of “a trashy and depraved type” [2]. However, he is financially successful through marriages and clever business deals. He has three sons from two previous marriages, Dmitri, Ivan, and Alyosha, plus an illegitimate son, Smerdyakov. All the brothers were abandoned to the care of relatives or strangers and essentially neglected by Fyodor Pavlovich. Most of the action takes place in the village of Staraya, Russia, the location of the family estate where all the brothers, now adults, have recently returned for various reasons.

The oldest brother and only child from the first marriage, Dmitri, 27, grew up believing that he had money and property from his mother, held in trust by his father, and that he would be financially independent on coming of age. Dmitri is a large, emotional, unruly man of “violent passions, impatient, and dissipated” [2]. He is convinced that his father has cheated him, and he intends to get his rightful inheritance, no matter what the cost. Besides the conflict over property and money, Dmitri is involved in a love triangle with his father over a beautiful young woman, Grushenka, who his father is trying to seduce with an offer of 3000 rubles cash. Dmitri is beside himself with jealousy and anger, and he had already physically assaulted his father.

The second brother Ivan, 24, is a sullen intellectual. Ivan is the prototypical rationalist who, like Dmitri, harbors deep hatred and anger for his father, but in a quiet, repressed way that is governed by his rationality. Ivan intellectualizes his emotions. All his acquaintances perceive him as cold and unfeeling. He flatly states that he does not believe in God or in immorality. His “facts-only”, hard-nosed approach to life precludes any close relationships.

The third brother, Alyosha, is a 20-year-old gentle, sensitive and compassionate man, the polar opposite of Ivan. He has spent the last year of his life in a monastery, not far from the Karamazov estate, developing his spirituality. He bears no ill will towards anyone, including his father. He has complete faith in God and in the people of the world. Since all the brothers have reunited, he has spent much of his time and energy trying to maintain the peace within the family.

The last brother, Smerdyakov is the illegitimate son of Fyodor. He is an unpleasant person who Fyodor verbally abuses and uses as his cook and lackey. Although Fyodor’s behavior is reprehensible, so many things about Smerdyakov are unsavory that one wonders why Fyodor ever admitted him into the household. Smerdyakov’s mother was a feeble-minded and homeless woman, living in the streets of Staraysa, raped by a drunken Fyodor Pavlovich on a dare from his friends. Smerdyakov is born in a bathhouse where his mother takes refuge and dies shortly thereafter. By chance, Fyodor’s childless servant, Grygory, discovers the baby, takes him into his cottage, and raises him out of the goodness of his heart. Unfortunately, none of that goodness rubbed off on Smerdyakov. Like Dostoevsky, Smerdyakov suffers from seizures, a condition associated with negative social stigmata during the nineteenth century.

The action accelerates when Dmitry starts nightly spying on his father, hoping to catch Grushenka going to visit Fyodor. Dmitry has sworn that he will kill his father if she goes to him. One night, Fyodor Pavlovich is brutally murdered, bludgeoned to death in his home, and the 3000 rubles he has put in an envelope for Grushenka is gone. That night Dmitry was again lurking in the garden, and he was spotted by the groundskeeper and mistaken as an intruder. Chased in the dark, Dmitry tries to flee, and when caught by the pants leg while attempting to scale the garden wall, Dmitry clubs the groundskeeper, causing a scalp laceration, and getting covered with blood in the process.

The groundskeeper calls the authorities, and later in the night, after the body of Fyodor is discovered, Dmitry is apprehended in the next town having fled with Grushenka. He is accused of the murder and brought before a judge and jury. The evidence points overwhelmingly to Dmitry as the killer; his need for money, the contested inheritance, his repeated threats to kill his father, the love triangle with Grushenka, the testimony of the groundskeeper, and the blood on his clothes. Despite all this evidence, Dmitry’s attorney, Fetyukovich, succeeds in planting a seed of doubt in the minds of the jurors, and the outcome of the trial begins to look more favorable for Dmitry until his spurned fiancé, Katerina, presents a letter from Dmitry declaring his murderous intentions.

The only other person to fall under suspicion was the illegitimate son, Smerdyakov, but he had a seemingly watertight alibi. He was having seizures during the night of the murder. A doctor and some of the investigators observed his violent convulsions during their investigation on the night of the murder, and the doctor testified that he thought the seizures so severe that Smerdyakov might not have survived the night.

Dmitry is convicted of patricide and sentenced to a life of hard labor in Siberia. Eventually, we find out that Smerdyakov was the actual killer. It turns out that when the investigators and the doctor observed Smerdyakov on the night of the murder, he was having what would be called today a psychogenic non-epileptic seizure, i.e., he was pretending.

Subsequent events do not turn out well. Dmitry labors away in Siberia. Ivan goes insane for his indirect role in the murder. Finally, Smerdyakov commits suicide, thereby eliminating any possibility of being discovered as the actual killer of his hated father and guaranteeing the blame for the murder remains with Dmitry, his despised half-brother. Smerdyukov’s psychogenic non-epileptic seizure was the perfect alibi to get away with patricide.

Dostoevsky

Fyodor Mikhailovich Dostoevsky was born in Moscow on October 30, 1821, and died in St. Petersburg on January 20, 1881, at the age of 59. He was the second child in a family of eight children. His mother was a quiet and pious woman who died of tuberculosis at the age of 37. His father was a military surgeon and a violent alcoholic who was gloomy and irritable. It was rumored that the family serfs murdered his father. This suspicious death haunted Dostoevsky all his life, and its influence can be seen in his novels.

Dostoevsky was a chain smoker, a heavy drinker and a compulsive gambler who had a “system” that, of course, did not prevent him from suffering massive losses. After many years of borrowing, pawning, and describing himself as a “dissolute, low, petty gambler,” he finally gave it up in 1871 [3].

Dostoevsky’s literary genius became apparent in 1846, when he was 24, with the publication of his first novel, Poor Folk. Publication of this novel introduced Dostoevsky to a vibrant literary and socialist group in St. Petersburg. After the tumult of the 1848 revolutions, Tsar Nikolai took a hard-line stance against the socialists, arresting and summarily executing many. Dostoevsky was arrested as a member of the Petrashevsky circle. He was sentenced to death, subjected to a frightening mock execution, but eventually given a sentence of four years hard labor in Siberia to be followed by a lifetime of service as a common soldier. He survived the hard labor, but his health deteriorated in Siberia. After a brief stint as a private in the army, he won political favor and was advanced to an officer’s rank and given his freedom. He soon resigned because of “ill health,” attaching a certificate from a physician attesting to his “falling sickness” with “attacks” that lasted ~15 minutes. This was the first documentation of Dostoevsky’s seizure disorder.

The nature of Dostoevsky’s epilepsy has been a controversial topic among “literary diagnosticians.” All authors agree that he did have a seizure disorder. Enough testimonials survive, including descriptions by friends, family, and the author himself, to verify the diagnosis of epilepsy. For instance, Major Ermakov in a Russian army report of December 1857 said, “Ensign Dostoevsky experienced an epileptic seizure with a sudden scream, loss of consciousness, convulsions of face and extremities, frothing at the lips, and respiratory rales” [4]. Furthermore, Dostoevsky may have had an inherited form of epilepsy since his father was said to have had attacks, and his son Alyosha died of status epilepticus at the age of 3 years.

Dostoevsky’s lifestyle may have contributed to the frequency and intensity of his seizures. Typically, he slept during the day and worked from 10:30 PM to 5:00 AM, drinking large amounts of tea and several glasses of sherry during the night. Also, his seizure frequency and intensity seemed to be related to psychological stress, and his alcohol abuse may have aggravated the condition.

Sigmund Freud (1856 - 1939) wrote a case history on Dostoevsky in 1928. He argued that Dostoevsky suffered from hysterical seizures due to the guilt surrounding the death of his father. “Now it is highly probable that this so-called epilepsy was only a symptom of his neurosis and must accordingly be classified as hystero-epilepsy” [5]. Freud was suggesting that Dostoevsky had pseudo-seizures! Modern authors reject Freud’s diagnosis, and I believe Dostoevsky did have epilepsy because the attacks occurred at night and during sleep, he sustained physical injuries from the attacks, and he clearly described post-ictal confusion, drowsiness and disturbances of speech. “Severe attack at 8:45 am: thoughts fragmentary, moving into other years, dreamy states, pensiveness, guilt. Dislocated disk in my back or damaged a muscle” [6].

Different authors have used different terms to classify Dostoevsky’s epilepsy. Henri Gasteau (1915 - 1995), the famous French neurologist, initially proposed a diagnosis of generalized convulsive seizures and later added the component of a temporal lobe lesion [7]. Voskuil [8] opined that Dostoevsky had partial complex epilepsy with a majority of secondary generalized nocturnal attacks and ecstatic auras. Kiloh [9] suggested a diagnosis of limbic epilepsy, and Baumann et al. thought he had mesial temporal lobe epilepsy [10]. Some of the contemporary descriptions of Dostoevsky’s seizures, and some of Dostoevsky’s descriptions of seizures in his novels, are incompatible with today’s nosology [11]. Although he no doubt had epilepsy, it appears that he took literary license with the descriptions of his fictional characters’ seizures and also with some of his own sufferings when writing to friends and relatives.

Dostoevsky had little respect for mid-nineteenth century physicians who had nothing to offer patients with epilepsy except their prevailing theory that seizures were due to excessive sexual excitement and masturbation. In 1846, Dostoevsky’s doctors treated him with leeches and two bloodlettings [8], both expensive treatments with no benefit. He watched helplessly as his son Alyosha died of status epilepticus. One can understand Dostoevsky’s negative feelings about his physicians. “How then can they be useful to humanity? They study only just enough to get paid appointments as soon as possible” [8].

Psychogenic non-epileptic seizures

Epilepsy comes from the Greek epilepsia meaning “to take hold of” or to “seize” as in being possessed or seized by demons. A better understanding of epilepsy came with the emergence of neurology as a science in the mid-nineteenth century. In 1873, the London neurologist John Hughlings Jackson (1835 - 1911) proposed that seizures were due to discharges of electro-chemical energy in the brain. Soon thereafter, potassium bromide was introduced as the first effective medication in the management of epilepsy, but there is no evidence that Dostoevsky used it. During the 1920s Hans Berger (1873 - 1941) developed the electroencephalograph, describing the alpha wave rhythm and confirming abnormal electrical discharges associated with the seizures. The release of phenobarbital and then phenytoin (Dilantin) in 1938 ushered in the modern era of anticonvulsants. Throughout much of recorded history, epilepsy produced fear in those having and in those observing a seizure. The negative social stigma surrounding epilepsy resulted in feelings of shame and depression, feelings expressed by Dostoevsky, “frightened my wife to death and filled me with melancholy and despondency” [9].

Psychogenic non-epileptic seizures (PNES) are observable, abrupt changes in behavior or consciousness that resemble an epileptic seizure, but the patient has no abnormal electrophysiological changes in the brain. The Diagnostic and Statistical Manual for Mental Disorders, DSM-5-TR classifies PNES as a conversion disorder with seizures. Some data indicate that 20% of the patients referred for epilepsy surgery and 50% of patients with refractory epilepsy have PNES [12]. Patients with PNES can have unconscious motivations, or they can be malingering and using the seizure for deception, as with Smerdyakov in the novel. About 15% of the patients with PNES also have a true seizure disorder, again as was the case with Smerdyakov. DeToledo notes that Dostoevsky had a clear understanding of the secondary gains derived from simulated seizures [13], and he used this understanding well in the novel.

Patients with PNES have been described as having a coping style characterized by hostility, anger, and mistrust, a description that certainly fits Smerdyakov [14]. Primary or secondary gain is a known factor behind PNES. Some patients with epilepsy can willfully alter neuronal activities in brain areas surrounding the epileptogenic focus and induce a real seizure [15]. Serdyukov tells Ivan that the morning after he murdered Feodor Pavlovich, he had a real seizure, perhaps brought on by the stress of the murder and the effort to bring off his own PNES.

Smerdyakov fools his adoptive parents, the village doctor, the investigators, the prosecutor, and the town’s people with his PNES. Only after his discussions with Ivan do we understand the true nature of Smerdyakov’s seizures and his crime. He had killed his father Fyodor Pavlovich, he let the blame fall on his hated older brother Dmitri, and he drove the other brother Ivan to madness. By hanging himself on the evening before Dmitry’s trial, Smerdyakov gets away with the perfect murder and one of the most fantastic revenge plots in literature.

The Brothers Karamazov

Readers have long discovered that Dostoevsky’s characters showcase his deep understanding of natural psychology and psychopathology, well before the codification of behavioral science. In The Brothers Karamazov, the author has exaggerated the strengths and weaknesses of his characters to illustrate various components of the human psyche. Dmitry is an example of the sensual: debauching, drinking, aggressively hostile, and spending lavishly. Ivan is pure intellect: rational, withdrawn, and isolated with no moral scruples as guideposts to life. Alyosha is the essence of spirituality: innocent, vulnerable, and with a particular concern for all children. Smerdyakov represents the evil that Dostoevsky thought lies latent within all humans.

Dostoevsky believed that evil resides in adults while children are pure and innocent until they become corrupted by the imperfect world in which they reside. Only after this corruption does the person have the capacity to inflict intentional suffering on another person which is the hallmark of evil for Dostoevsky. As to how a life should be lived, Smerdyakov demonstrates that hate is a form of self-immolation. Ivan shows that the intellect without the spirit is a dead end. Dmitry and Fyodor show that life is more than objects, property, and money, all of which are meaningless without love. Alyosha teaches us that children and love are the salvation of life and the hope for the future. Dostoevsky believed that to help a child was to experience the divine [1].

## Conclusions

A century and a half after his death, Fyodor Mikhailovich Dostoevsky continues to offer us valuable insight into the human psyche and interpersonal relationships. At first glance, it seems unfortunate that epilepsy hung over his life like the sword of Damocles. As for Dostoevsky, we can only speculate how his life and his creativity would have been different if his doctors in 1865 could have written a prescription for him to take a 100 mg capsule of Phenytoin three times a day.
